# Supplementary material for: Estimating the optimal interval between rounds of indoor residual spraying of insecticide using malaria incidence data from cohort studies
Source: PLoS One. 2020 Oct 23;15(10):e0241033. doi: 10.1371/journal.pone.0241033 (PMC7584202; doi:10.1371/journal.pone.0241033)
Supplement: Attachment — Submitted filename: Plos One comments 19032020PONE-D-20-03773.docx [file pone.0241033.s003.docx]

**General comments**

This article is timely and addresses a very important neglected component of IRS that can effectively improve its impact, and it is definitely worth publishing and communicating the information to the implementing programs. Even though the author has addressed most of the relevant points, the following aspects, comments and questions need clarification before considered for publishing this article.

**Introduction:**

- The authors have explained that IRS has been shown to be effective in terms of reducing malaria incidences and entomological indicators in various settings. However, in several other studies conducted from other endemic settings across Africa, contrasting results have also been documented, and this does not devalue the importance of IRS. So I suggest to the authors to include the two contrasts in their paper and perhaps, as timing could partly play a role in the negative outcomes reported in other endemic settings alongside other factors.
- The authors have indicated factors that in general may affect the overall effectiveness of IRS such as individual insecticides, operational factors etc., however, one of the key indicator or factor crucial to the effectiveness of IRS is compliance even though extremely neglected for IRS, which is basically protecting the personal household space after IRS implementation to ensure high effectiveness of the product. I also suggest here to the authors to include human behavioural factor as various activities conducted by household owners within personal household spaces may greatly affect the IRS efficacy and hence needs to be considered as a key component while planning for IRS implementation periods.

**Methodology:**

- LLINs were distributed almost less than 2 months before IRS implementation, in 2017, and given that nets (pyrethroid nets) have been shown to repel or irritate mosquitoes, is there any masking effect that may have led to the assumption that incidence greatly reduced as a result of IRS?
