# Supplementary material for: Estimating the optimal interval between rounds of indoor residual spraying of insecticide using malaria incidence data from cohort studies
Source: PLoS One. 2020 Oct 23;15(10):e0241033. doi: 10.1371/journal.pone.0241033 (PMC7584202; doi:10.1371/journal.pone.0241033)
Supplement: Attachment — Submitted filename: Review_PLOS _2020.docx [file pone.0241033.s005.docx]

**Manuscript**: Estimating the Timing Interval for Malarial Indoor Residual Spraying: A Modelling Approach

The authors provided some interesting data for effective use IRS in malaria control, thought there are issues that need attention

One is the cost effectiveness of the repeated application of the IRS, which is also directly related with the sustainability of the program

Another one is that the authors indicated the malaria incidence at baseline month, midline month and endline month, but not in between. Fig 1 shows some interesting results—where more malaria cases are observed immediately after IRS of both insecticides.

What would happen if the first application miss correct timing—which month/or season is suitable for the first IRS? The first IRS timing not affect the results?

What would happen if the actellic insecticide is the only insecticide applied throughout the study period? --- 40 weeks intervals still works? What about the bediocarb alone?

The authors didn’t indicate the impact of bed nets independently? Bed nets could affect the interval?

Fig 2 and 3---hard to understand— possible to simplify

**Methods:** Was it systematically designed to answer the question? For example – the number of children in phase 1 and 2 are different? This is weakest part of this study, and lead to the results of inconclusive.

Discussion: ….The finding on increase in malaria incidence after IRS application is similar to what other authors reported. For example, other studies in Uganda documented increases in malaria incidence and test positivity rate (TPR) post IRS…. **not justifiable results**

This result need another justification ….method section and the initial spray time

Conclusions: there are a lot of uncertainty to reach these conclusions

Finally, major revision is required if considered for publication
