# Supplementary material for: Estimating the optimal interval between rounds of indoor residual spraying of insecticide using malaria incidence data from cohort studies
Source: PLoS One. 2020 Oct 23;15(10):e0241033. doi: 10.1371/journal.pone.0241033 (PMC7584202; doi:10.1371/journal.pone.0241033)
Supplement: Attachment — Submitted filename: Response to Reviewers.docx [file pone.0241033.s006.docx]

**Reviewer #1**

**General comments**

This article is timely and addresses a very important neglected component of IRS that can effectively improve its impact, and it is definitely worth publishing and communicating the information to the implementing programs. Even though the author has addressed most of the relevant points, the following aspects, comments and questions need clarification before considered for publishing this article.

**Introduction:**

- The authors have explained that IRS has been shown to be effective in terms of reducing malaria incidences and entomological indicators in various settings. However, in several other studies conducted from other endemic settings across Africa, contrasting results have also been documented, and this does not devalue the importance of IRS. So I suggest to the authors to include the two contrasts in their paper and perhaps, as timing could partly play a role in the negative outcomes reported in other endemic settings alongside other factors.

**Response**: Thank you so much for this suggestion. The following two sentences have been added in the introduction at the bottom of the first paragraph. Lines 60-62 of manuscript. “However, some non-pyrethroid IRS products have contrasting impacts(8). For example, a study in Benin, West Africa shows that short lasting efficacy of Actellic 50 EC may render IRS ineffective(9).”

- The authors have indicated factors that in general may affect the overall effectiveness of IRS such as individual insecticides, operational factors etc., however, one of the key indicator or factor crucial to the effectiveness of IRS is compliance even though extremely neglected for IRS, which is basically protecting the personal household space after IRS implementation to ensure high effectiveness of the product. I also suggest here to the authors to include human behavioural factor as various activities conducted by household owners within personal household spaces may greatly affect the IRS efficacy and hence needs to be considered as a key component while planning for IRS implementation periods.

**Response**: Thank you for this suggestion. The following sentence has been added in the introduction in the second paragraph. Lines 65-67 of manuscript. “The other factor which is often neglected is compliance by household owners to protect household space after IRS implementation to ensure high effectiveness.”

**Methodology:**

- LLINs were distributed almost less than 2 months before IRS implementation, in 2017, and given that nets (pyrethroid nets) have been shown to repel or irritate mosquitoes, is there any masking effect that may have led to the assumption that incidence greatly reduced as a result of IRS?

**Response**: Thank you. We agree with you that LLINs can modify the effect of IRS. We have added the following sentence under methodology at the bottom of the study setting paragraph. Lines 134-135 of manuscript. “Studies have shown an additional effect of LLINs over IRS(18), meaning the effect of IRS could have been modified by use of LLINs.”

**Reviewer #2**

**Manuscript**: Estimating the Timing Interval for Malarial Indoor Residual Spraying: A Modelling Approach

The authors provided some interesting data for effective use IRS in malaria control, thought there are issues that need attention

One is the cost effectiveness of the repeated application of the IRS, which is also directly related with the sustainability of the program

**Response:** We agree with the reviewer regarding the cost of IRS and in this paper we recommended that while our results suggest shorter intervals than the usual practice, the cost of IRS should be considered. Indeed, cost-benefit analysis should be exercised before considering our recommended intervals. No changes made to the paper.

Another one is that the authors indicated the malaria incidence at baseline month, midline month and endline month, but not in between. Fig 1 shows some interesting results—where more malaria cases are observed immediately after IRS of both insecticides.

**Response:** Thank you for this observation. Estimates at baseline, midline and endline simply provide snapshots in trend for malaria incidence but a full profile is given in Fig 1. No changes made to the paper.

What would happen if the first application miss correct timing—which month/or season is suitable for the first IRS? The first IRS timing not affect the results?

**Response**: We agree with the reviewer that poor timing of the first round of IRS can affect its effectiveness and our estimates. To account for this, we performed a sensitivity analysis of time-lag in rainfall whereby a 2-months lag offered a better fit. No changes made to the paper.

What would happen if the actellic insecticide is the only insecticide applied throughout the study period? --- 40 weeks intervals still works? What about the bediocarb alone?

**Response**: If actellic is the only insecticide applied, we would recommend an interval shorter than 40 weeks after the first round. A study in Zanzibar to investigate the residual efficacy of actellic sprayed on common surfaces of human dwellings showed that efficacy of actellic 300CS was maintained on all sprayed surfaces up to 8 months (32 weeks) post-IRS (1). If only bendiocarb, then our estimated intervals would apply. However, our results suggest that the timing differs for subsequent rounds. No changes made to the paper.

The authors didn’t indicate the impact of bed nets independently? Bed nets could affect the interval?

**Response**: We did not study the impact of bed nets independently. Yes, bed net use could affect the IRS interval. In our study setting bed nets were distributed to participants at enrollment and a universal distribution in May 2017 which means that our estimates could be modified by bed net use. To Acknowledge bed net contribution, the following sentence has been added under methodology. Lines 134-135 of manuscript. “Studies have shown an additional effect of LLINs over IRS(18), meaning the effect of IRS could have been modified by use of LLINs. A seond limitation has been added under limitations by adding the following two sentences. Lines 259-262 of manuscript. “Secondly, all participant received a bed net at recruitment and there was a mass net distribution campaign in 2017 and the observed IRS impact may have been affected by the delivery of nets. With this, the results observed should be more applicable in settings where IRS is implemented with high bed-nets coverage.”

Fig 2 and 3---hard to understand— possible to simplify

**Response**: Fig. 2 are diagnostic plots showing how well the GAM model fitted the data. The following statement has been added to the figure’s legend for clarification. Line 206 of manuscript. “A straight horizontal line through point 0 on the y-axis implies better fit.”

Fig. 3 shows both observed (blue points) and predicted (solid red line) incidence of malaria with monthly rainfall superimposed as orange vertical bars stratified by round of IRS. The secondary y-axis labelled in orange represents the amount of monthly rainfall in mm. To further simply the figure, each axis for each panel figure has been labelled. We have also revised the legend of Fig. 3 to read as follows (see edits highlighted in yellow). Lines 221-225 of manuscript.

“Fig 3. Observed (blue points) and predicted (solid red line) incidence of malaria per person-years against time in weeks since application of IRS and monthly rainfall (vertical orange bars) against calendar time for each round of carbamate bendiocarb (top row) and actellic (bottom row). The secondary y-axis labelled in orange represents the amount of monthly rainfall in mm. The vertical dotted lines indicate optimal time for applying another round of IRS. ”

**Methods:** Was it systematically designed to answer the question? For example – the number of children in phase 1 and 2 are different? This is weakest part of this study, and lead to the results of inconclusive.

**Response**: The study was not designed to answer the question. We acknowledged this limitation and recommended in our conclusion that “timing and incidence should be monitored in the future to improve these estimates.” A limitation of this has been included by adding the following two sentences. Lines 262-263 of manuscript. “Thirdly, the study was not designed to estimate the timing interval for IRS. A future study designed to estimate the timing interval for IRS could help improve our estimates.”

Discussion: ….The finding on increase in malaria incidence after IRS application is similar to what other authors reported. For example, other studies in Uganda documented increases in malaria incidence and test positivity rate (TPR) post IRS…. **not justifiable results**

This result need another justification ….method section and the initial spray time

**Response**: Thank you for this observation. The following sentence has been added to the discussion at the end of the 3^rd^ paragraph. Line 257 of manuscript. “This could be due to poor timing of IRS.”

Conclusions: there are a lot of uncertainty to reach these conclusions

**Response:** The conclusions made here are subject to the study limitations highlighted in the paper such as the inability to generalize the results. We recommended that timing and incidence be monitored in future to refine our results.

Finally, major revision is required if considered for publication

**Response**: The necessary revisions have been made

1.	Haji KA, Thawer NG, Khatib BO, Mcha JH, Rashid A, Ali AS, et al. Efficacy, persistence and vector susceptibility to pirimiphos-methyl (Actellic&reg; 300CS) insecticide for indoor residual spraying in Zanzibar. Parasites and Vectors. 2015;

**Response**: The suggested reference has been added, see reference number 12, line 305 of manuscript.

**Reviewer #3**

Article: PONE-D-20-03773

Title: Estimating the timing interval for malarial indoor residual spraying: a modelling approach

Summary: This is an interesting analysis of clinical incidence data tracking a cohort of under 10-year olds living in a high transmission setting that has implemented IRS. During Phase I, the study explores the use of bendiocarb sprayed approximately bi-annually. During phase II, the study explores annual implementation with Actellic 300®CS. The analysis is robust, and this is an interesting method to determine the window between spray rounds. However, I have some major concerns for the authors to consider prior to acceptance for publication.

Major concerns

1. The authors do not mention the natural seasonality of mosquitoes, how their density may change in the absence of chemical intervention, or whether these changes subsequently drive any seasonality in clinical incidence. Please could the authors comment on how this was accounted for in the statistical model as an increase in cases (the optimal point) could still indicate a working IRS product if the cases increase at a slower rate than expected in the absence of said product at specific times of the year. Were there any data on mosquito vector densities to corroborate the impact on incidence is driven by the IRS impact?

**Response**: The analysis did not directly include natural seasonality of mosquitos, however, we included the amount of monthly rainfall in the model which turned out insignificant and was dropped from the final fit. While including the monthly rainfall, a sensitivity analysis was conducted considering different lags until a better fit was attained. Usually, the mosquito densities vary with the amount of rainfall. No changes made to the paper.

2. How was clinical incidence monitored in the trial? Were rapid diagnostic tests used to confirm positivity? This is important because if the presence of fevers are used to indicate malaria positivity (the authors note symptoms, but do not specify what these are for this study) then ‘true’ seasonality may be dampened because of other infections causing fever that are mis-diagnosed to be malaria. Please comment to clarify.

**Response**: In both phases of the study, malaria positivity was confirmed using microscopy. The following sentence has been added under study population section. Line 144 of manuscript. “Clinical malaria was defined as having fever and a positive blood smear.”

3. As all participants also received an ITN on enrolment, how were the impacts of these interventions decoupled from the impact of the IRS products? How were the effects adjusted for later rounds of IRS when the ITN efficacy would have waned and adherence to ITN use may have diminished?

**Response**: Thank you for this observation and we agree with you that ITN use could have affected our estimates. We have added a limitation as follows. Lines 259-261 of manuscript. “Secondly, all participants received a bed net at recruitment and there was a mass net distribution campaign in 2017 and the observed IRS impact may have been affected by the delivery of nets. With this, the results observed should be more applicable in settings where IRS is implemented with high bed-nets coverage.”

In addition, were ITNs redistributed at any point through the study? If not, is the impact from Actellic even greater because it acts with older ITNs – potentially used less by the community and almost certainly working less effectively because the active ingredient has waned. It is critical to comment or account for this effect.

**Response**: There were ITNs distributed at enrolment of the study participants and there was a universal distribution of LLINs in May 2017 during the study period when actellic was applied. Yes, this could have impacted on our estimates for intervals of actellic and this has been acknowledged by adding the following sentence under discussion. Lines 247-249 of manuscript. “Though actellic is known to be longer-acting than bendiocarb, the estimated longer intervals for the former could partly be due to the effect of universal distribution of LLINs which occurred in May 2017.”

4. Given that malaria cases are so sensitive to the local ecology for any given transmission season, could the authors comment on the uncertainty in their suggested interval for spray rounds and whether they can infer anything from the statistical model about how this window may vary season to season, year to year or across any other boundary. Is it possible to make any sort of prediction for other settings using this framework? The authors note that uncertainty will be considered in future work but this should be included here. Could you infer uncertainty from the 90% credible intervals of the Bayesian simulations carried through from the splines? Or use the mean +/- 1.96*SE to estimate some uncertainty for the spline predictions shown in Fig 3?

**Response**: Seasonality was accounted for by including lagged monthly rainfall into the model, though this was not significant and it was dropped from the final fit. Our results were limited to one site making generalizability of the results to other settings quite infeasible. This was mentioned in the limitations of the study. Uncertainty is now accounted for by adding 95% confidence intervals for the timing intervals. See lines 213-215 of manuscript.

Is there any sense in fitting the estimate across rounds for each chemistry? Rather than after each round separately to try and infer a more generalised estimate for this interval between spraying? Could the authors source data from other sites to refit the model with additional data which would enable the uncertainty to be better captured and results to be generalised which would be more useful for guiding policy decisions.

**Response**: We did not have access to additional data from other sites as this was the only site in Uganda where cohort studies were in place to measure malaria incidence. We have already stratified the analysis by IRS round and chemistry (bendiocarb vs. Actellic) and we think this is sensible. No changes made to the paper.

Minor suggestions

The title might be more informative, perhaps try:

“Estimating the interval between indoor spraying of residual chemistries from clinical incidence data”

“Optimising the interval between residual spraying of insecticides indoors in a high transmission setting”

The term ‘modelling’ is fairly broad so I think the title could better reflect the work if this were altered.

**Response**: Thank you so much for your suggestions. We have revised the title to read as “Estimating the optimal interval between rounds of indoor residual spraying of insecticide using malaria incidence data from cohort studies”. See lines 1-3 of manuscript

Line 98: ‘diving’ should be ‘dividing’

**Response**: Done. Line 103 of manuscript

Line 221: ‘long-acting’ should be ‘longer-acting’

**Response**: Done, Line 230 of manuscript

Line 230 and 231: should read ‘concurs’ and ‘concur with’ not ‘conquers’ or ‘conquer with’

**Response**: Done. Line 239 of manuscript
