# Supplementary material for: Estimating the optimal interval between rounds of indoor residual spraying of insecticide using malaria incidence data from cohort studies
Source: PLoS One. 2020 Oct 23;15(10):e0241033. doi: 10.1371/journal.pone.0241033 (PMC7584202; doi:10.1371/journal.pone.0241033)
Supplement: Attachment — Submitted filename: Response to Reviewers.docx [file pone.0241033.s007.docx]

August 21, 2020

To: The Editor-in-Chief

PLOS ONE

Reference number: PONE-D-20-03773

Thank you very much for the thoughtful reviews of our manuscript and for inviting us to re-submit our revised manuscript (based on the reviewers’ comments) titled: “Estimating the Optimal Interval between Rounds of Indoor Residual Spraying of Insecticide Using Malaria Incidence Data from Cohort Studies”. Below are point by point responses to the reviewers’ comments. We confirm that the material in this manuscript has not and will not be offered elsewhere for possible publication, as long as it is under PLOS ONE consideration. We look forward to your favourable consideration of this re-submission.

POINT BY POINT RESPONSES TO THE REVIEWERS’ COMMENTS.

**Reviewer 1:**

Review
Article: PONE-D-20-03773R1
Title: Estimating the Optimal Interval between Rounds of Indoor Residual Spraying of Insecticide Using Malaria Incidence Data from Cohort Studies

Summary: The work tracks clinical incidence data for a cohort of 6-month to 10-year olds living in a high transmission setting that has implemented IRS. During Phase I, the study explores the use of bendiocarb sprayed approximately bi-annually. During phase II, the study explores annual implementation with Actellic 300®CS. The limitations of the study are better explained in this revision. However, I still have some major reservations.

There remain a few queries on the methods and I have some hesitations on how the results are discussed – particularly in the abstract

Thank you for providing the data, that is useful for the review process. How was the annual EIR calculated? Please add a few sentences to explain how this was done with the data available. Similarly, it looks like rainfall was estimated monthly or over longer periods – is it possible to get finer scale information on rainfall – by week perhaps? This could impact dramatically on whether it is statistically important.

**Response**:

Annual EIR defined as the number of infectious bites per person per year was calculated as a product of the daily human biting rate (HBR) and the sporozoite rate × 365 days/year. The following sentence has been added in the methodology “EIR was calculated as a product of the daily human biting rate (HBR) and the sporozoite rate × 365 days/year as previously described by our group (16)”. See lines 135-136 of the manuscript.

Unfortunately, rainfall data was received when already aggregated by month and therefore we could not revise the results using weekly data. A discussion of this has been added as follows “For rainfall and EIR, perhaps it is because the data we used were already aggregated by month. Finer intervals like weekly data would help to improve our estimates.” See lines 272-274 of the manuscript.

The methods first focus on the statistical methods, but it may be more intuitive to present the study area, data collection and PRISM trial prior to the statistics to help the reader follow the work reported.

**Response**: This has been revised as suggested. See lines 96-161 of the manuscript.

Have you tried to include a variable like ‘month since net distribution’ or even a binary ‘net distribution within 1 year’ variable to try to account for the distinct impact seen from new nets?

**Response**: A variable for months since net distribution has been included in the analysis. However, this did not improve the model fit (p=0.249). Note that nets were distributed at enrolment and there was a universal distribution in May 2017. This result has been presented. See lines 200-201 of the manuscript.

The authors present, in the abstract, the clinical incidence from October 2014 and February 2019. But this could be driven by seasonality. It would be better to compare two times from the same month of the year, or even better still, averaging clinical incidence across a wider window of time to mitigate somewhat for natural between-year stochasticity in the metric. For how many months were the children tracked prior to application of the spray? Could you average across this entire period and compare it to the same length of time (and months) in the post application periods? Can you also provide the baseline data in the supplement?
I have the same issue with Table 1.
Table 1 is not so useful as it currently stands – it could be more informative to average or sum clinical incidence in the tracked cohorts across longer time windows (matching months) as, although seasonal rainfall did not appear to be associated with the duration of IRS effectivity in the GAM analysis, seasonal patterns in malaria incidence are likely and do tend to drive transmission by increasing the EIR, so comparing different times of the year may be misleading. Again, averaging or incidence over 3 or 6 months at baseline and comparing that to the same months in later years would be a fairer presentation. This seems particularly important because Fig 1 seems to suggest a higher mean incidence in Oct 2016 (than the baseline Oct 2014 estimate) but low in Dec 2016 – which is chosen as the comparison estimate … this is not a fair comparison so estimating the average clinical incidence across multiple months to some extent could overcome this potential stochasticity in measurements and alleviate the need to arbitrarily select a single measurement for the table.

**Response**: Thank you so much for this observation and for the suggestions. We have revised the presentation of our results in Table 1 by looking at the 6-months period before IRS (baseline) compared to the 6-months periods post IRS application. The periods we considered are Nov2013-Apr2014, May2014-Oct2014, Nov2014-Apr2015, May2015-Oct2015, Nov2015-Apr2016, May2016-Oct2016, Nov2016-Apr2017, May2017-Oct2017, Nov2017-Apr2018 and May2018-Oct2018. The incidence declined from 2.96 per person years during the baseline period (May-October 2014) to 1.74 during the first period following the first round of IRS application (November 2014-April 2015) and then to 0.02 during the last period following the 6th round of IRS. See lines 177-186 of the manuscript.

I have also a suggestion for the abstract as I think that the methods/results and particularly conclusions paragraphs could be more useful if altered slightly.
• Use the IRS product (presumably Ficam® and Actellic 300®CS) or the insecticide active ingredient (bendiocarb and pirimiphos methyl) not one of each [this issue applies throughout the manuscript]

**Response**: Thank you for this observation. Consistency has been obtained throughout the manuscript by using insecticide active ingredient names.

• Present the empirical data first, then the statistics

**Response**: Empirical data is now presented before statistics. See line 37 of the manuscript.

• Refer to the model as a statistical approach (as it is not a predictive model as the mechanism of transmission is not determined), so statistical approach is clearer.

**Response**: This has been revised as suggested. See lines 29 and 50 of the manuscript.

• My greatest hesitation is with the conclusion. Here, a statistical model is applied which is useful to capture the direction of an effect or compare the two IRS products, but it is not useful for future projections because it does not capture the mechanism of impact. So, inferring from the statistical model needs to be done with care. Drawing contrasts between the spray applications is reasonable, but perhaps soften how this is stated so as not to determine future application.
Below is a suggestion using tracked changes [comments to address/delete!]:
“Background : Indoor residual spraying (IRS) reduces vector densities and malaria transmission, however, the most effective spraying intervals for IRS have not been well established. We estimated the optimal timing interval for IRS using a statistical approach.
Methods : Six rounds of IRS were implemented in Tororo District, a historically high malaria transmission setting in Uganda, during the study period (3 rounds with Ficam (bendiocarb active ingredient): December 2014 to December 2015, and 3 rounds with Actellic 300®CS (pirimiphos methyl active ingredient): June 2016 to July 2018). Generalized additive models were used to estimate the optimal timing interval for IRS based on the predicted malaria incidence. The model was fitted to clinical incidence data from a cohort of children aged 0.5–10 years from selected households observed throughout the study period. Annual entomological inoculation rates were estimated [how] and aggregated monthly [looking at the data file it looks like these were averaged?] .
Results : Monthly incidence of malaria from October 2014 to February 2019 decreased from 3.25 to 0.0 per person-years in the children under 5 years, and 1.57 to 0.0 for 5-10 year-olds [this is not appropriate, please see comments above]. The optimal time interval for IRS differed between bendiocarb and pirimiphos methyl and by IRS round. To retain an optimum impact, Ficam® would require respraying 17 weeks after application whereas Actellic 300®CS could remain impactful for 40 weeks, although in the final year this estimate 36 weeks. However, we could not estimate from the data the optimal time after the second and third rounds of bendiocarb and after the second round of actellic. Neither the amount of rainfall nor the EIR were found to be statistically significant for determining the time period between spray rounds [although these are aggregated by month(?) so the data may lack the scale needed to identify impact].
Conclusion: In our setting, the effect of the two IRS products was distinct. Statistically, Actellic 300®CS provided a longer window of protection than Ficam®, although impact varied between different spray rounds and years which was not explained by rainfall or EIR in our statistical approach. Understanding the effectiveness of IRS and how long it lasts can help for planning campaigns, but one should consider the financial cost and insecticide resistance. Monitoring the timing of spray campaigns using clinical incidence could be repeated in future programs to help determine the average period of protectivity of these products.”

**Response**: Thank you so much for these suggestions. The abstract has been revised using your suggested edits. However, we preferred using active ingredient names instead of trade names. See lines 27-53 of the manuscript.

There is no discussion on the timing of the intervention relative to the transmission peak. Generally, Uganda has a higher transmission peak in May/June time, so perhaps the November spray rounds have artificially bigger impacts due to fewer cases / less transmission in these periods of the year.

**Response**: Thank you so much for this observation. The following sentence acknowledging transmission peaks in the months of May-June has been added “*We also observe transmission peaks in the months of May and June which coincided with the second, fourth, fifth and the sixth rounds of IRS*.” See lines 190-192 of the manuscript.

A discussion on November spray rounds has been added in the discussion as a possible explanation for the increase in the incidence after IRS as follows: “*This could be due to poor timing of IRS relative to the transmission peak. For example, our results indicate higher transmission peak in May/June time, so perhaps the November spray rounds may have artificially bigger impacts due to less transmission in these periods of the year*”. See lines 267-270 of the manuscript.

Thank you for adding the sentence on fever and blood smear positivity. Just to clarify, did the blood smear have to be positive for a clinical case to be scored? Or were some patients classed to be malaria positive by fever only? This could alter assumptions.

**Response**: Yes, all malaria cases were first confirmed by a positive blood smear. A case of malaria was defined as having a fever (tympanic temperature > 38.0°C or history of fever in the previous 24 hours) and a positive thick blood smear was by light microscopy.

It may be worth including a sentence or two on what it means for the analysis to miss asymptomatic cases and what proportion of the population may be asymptomatic (a good paper on this issue e.g. Lindsay Wu et al Nature 2015: <https://www.nature.com/articles/nature16039?proof=true1>)

**Response**: Although the data on asymptomatic cases is available for this cohort, we used clinical malaria as the outcome as these are the individuals who would routinely be identified at the health facilities and are the main group that would drive policy decisions in an endemic setting. The prevalence of asymptomatic parasitemia was estimated at 58% in October 2014 (baseline) and 7% in February 2019 (end line). Details of the prevalence of parasitemia by age-group has been published elsewhere (Nankabirwa *et al.* 2020). The limitation section has been updated to capture this as follows. “*Thirdly, our analysis did not consider asymptomatic infections which could possibly alter our estimates. For example, in the study setting the prevalence of asymptomatic infection was estimated at 58% in October 2014 (baseline) and 7% in February 2019 (end line)(16)*”. See lines 278-281 of the manuscript.

**Reviewer 2:**

Additional Minor concerns:

I understand the author’s focus is on timing of IRS with benthiocarb and Actellic, however, I still think there is a missing link on how the author has addressed the question 1 linked to paragraph one in the introduction section. In my opinion not only non-pyrethroids have produced contrasting impacts.

**Response**: Non-pyrethroids has been dropped and the sentence now read as follows: “*However, some IRS products have contrasting impacts(8)*.” See line 64 of the manuscript.

In the methodology section, the author has addressed that studies have shown that LLIN may have effect yet only referenced 1 article. I also think the statement requires a bit of modification to suit its placement in the methodology section, otherwise the author has mentioned a similar comment in the discussion section, under limitation so either way do away with it in methodology section or rephrase.

**Response**: The manuscript has been revised: 1) to add new references that have assessed the impact of LLINs on malaria burden, 2) a variable looking at months since distribution of LLINs has been included in the model, although this did not improve the model fit (see lines 200-201 of the manuscript), 3). The statement on LLINs under methodology has been revised as follows “*Studies have shown an additional effect of LLINs over IRS(15). The effect of LLINs on the IRS timing interval has been assessed in this analysis.*” See lines 107-108 of the manuscript.

Reviewer #2: Please see the attached response to authors

I have indicated that not all data are provided but only because the baseline data are not shown and these would be useful. Thank you for providing the remaining data

**Response**: Baseline data is now analysed looking at 2 six-months periods before IRS (see Table 1). The remaining selected baseline data are shared.

I have some concerns on using the monthly (or longer) estimates of rainfall and EIR for associations with timing of spraying. This may not be a fine enough resolution to usefully determine an association and may explain why none were found.

**Response**: Thank you for this information, unfortunately the rainfall data available is already aggregated by month. We appreciate that this could be a long period to determine the associations with malaria incidence. This limitation has been acknowledged in the discussion as below: “*The findings show that the timing interval was not altered by the amount of rainfall, the entomological inoculation rate (EIR), and the duration in months from when nets were distributed. For rainfall and EIR, perhaps it is because the data we used were already aggregated by month. Finer intervals like weekly data would help to improve our estimates.” See lines 271-275 of the* manuscript.
