# Supplementary material for: Estimating the optimal interval between rounds of indoor residual spraying of insecticide using malaria incidence data from cohort studies
Source: PLoS One. 2020 Oct 23;15(10):e0241033. doi: 10.1371/journal.pone.0241033 (PMC7584202; doi:10.1371/journal.pone.0241033)
Supplement: Attachment — Submitted filename: Response to Reviewers.docx [file pone.0241033.s009.docx]

August 21, 2020

To: The Editor-in-Chief

PLOS ONE

Reference number: PONE-D-20-03773

Thank you very much for the thoughtful reviews of our manuscript and for inviting us to re-submit our revised manuscript (based on the reviewers’ comments) titled: “Estimating the Optimal Interval between Rounds of Indoor Residual Spraying of Insecticide Using Malaria Incidence Data from Cohort Studies”. Below are point by point responses to the reviewers’ comments. We confirm that the material in this manuscript has not and will not be offered elsewhere for possible publication, as long as it is under PLOS ONE consideration. We look forward to your favourable consideration of this re-submission.

POINT BY POINT RESPONSES TO THE REVIEWER’S COMMENTS.

Review
Article: PONE-D-20-03773R2

Title: Estimating the Optimal Interval between Rounds of Indoor Residual Spraying of Insecticide Using Malaria Incidence Data from Cohort Studies

Summary: The work tracks clinical incidence data for a cohort of 6-month to 10-year olds living in a high transmission setting that has implemented IRS. During Phase I, the study explores the use of bendiocarb sprayed approximately bi-annually. During phase II, the study explores annual implementation with Actellic 300®CS. The draft is improved on the previous versions and the previous comments are addressed well. I have a few minor comments to add and a couple of suggestions to pass on to the authors.

**Introduction**

There is a reference for the statement in lines 68-70: (<https://malariajournal.biomedcentral.com/articles/10.1186/s12936-020-3102-6>)

**Response**: Thank you so much for the suggested reference. This has been added. See lines 68-70

This sentence, starting line 70, is not quite correct as thatch is mentioned twice – please correct:

“A study in Zimbabwe showed that bendiocarb remains active for up to 8 weeks with 96% mortality for mosquitoes on thatch, and 20 weeks with 74% mortality for mosquitoes on mud compared to 100% for mosquitoes on thatch (10).”

**Response**: Thank you for this observation. The sentence has been revised to read as follows: “A study in Zimbabwe showed that bendiocarb remains active for up to 8 weeks with 96% mortality for mosquitoes on thatch(11). The same study showed that bendiocarb remains active for up to 20 weeks with 74% mortality for mosquitoes on mud compared to 100% for mosquitoes on thatch(11).” See lines 71-74

Similarly the following sentence needs to be adjusted slightly for clarity:

“While in Madagascar, bendiocarb was shown to maintain 80% mosquito mortality for up to 5 months post spraying (11).”

**Response**: The sentence has been revised to read as follows: “In Madagascar, bendiocarb was shown to have a mortality inducing effect on local mosquitoes of up to 80% for up to 5 months post spraying (12).” See lines 74-76.

Throughout this second paragraph of the introduction, efficacy is used without defining exactly what is meant. Perhaps state that a mortality inducing effect where more than 80% of susceptible or local mosquitoes are killed in a bioassay test on the sprayed surfaces is considered effective by WHO guidelines [ref]. (*In my opinion, if sprays are killing mosquitoes at a less potent level they may well still have robust impact on incidence – but this is not the WHO definition at the moment!)* Otherwise you could stick to quantifying this effect by stating that the mortality inducing effect on local mosquitoes remained above 80% for xx weeks as noted by the referenced studies.

**Response**: Thank you for this observation and suggestion. We are happy to consider your suggested revision by replacing efficacy with “mortality inducing effect on local mosquitoes”. The affected sentences have been revised to read as follows: “In Madagascar, bendiocarb was shown to have a mortality inducing effect on local mosquitoes of up to 80% for up to 5 months post spraying (12). Elsewhere in Zanzibar, a study to investigate the residual effect of pirimiphos methyl active ingredient sprayed on common surfaces of human dwellings showed that its mortality inducing effect on local mosquitoes was maintained on all sprayed surfaces up to 8 months post-IRS (13).” Lines 74-78.

One interesting part of your study is that your tested outcome is a clinical one. Testing IRS on mosquito mortality alone forces us to make an assumption about how reduced mosquitoes may lead to reduced clinical incidence. Your approach allows you to test the durability of the spray campaigns on the clinical outcome. I think this is worth highlighting, either as a sentence in the introduction or discussion.

**Response**: Thank you for this important suggestion. The following sentence has been added in the last paragraph of the introduction “The use of incidence data allows us to test the durability of the spray campaigns on the clinical outcome.” Lines 90-91.

It is also worth noting that malaria transmission in Uganda is mostly perennial, so even though there are slight seasonal peaks, transmission continues all year making the durability of the IRS product an important consideration. This helps understand the analysis too because the 6-month periods can be more consistently compared than would be the case in an area that had more seasonal transmission.

**Response**: This is a true observation. A sentence noting this has been added under the study setting as follows: “Malaria transmission in Uganda is mostly perennial, so even though there are slight seasonal peaks, transmission continues all year making the durability of the IRS product an important consideration.” Lines 103-105.

**Discussion**

Line 259 should be ‘concurs’ not ‘concur’

**Response**: This is done. The sentence now reads as follows: “However, for pirimiphos methyl, the results suggest that about one round per year is feasible and this concurs with the WHO recommendations(14).” See lines 263-265.

On line 264 perhaps say ‘may leave communities unprotected’ rather than may render IRS ineffective

**Response**: This is done. The sentence now reads as follows: “This implies that waiting for 7 months in case of bendiocarb before another round of IRS may leave communities unprotected.” See lines 268-269.

Lines 269- Perhaps say ‘due to the natural decline in transmission observed during these months of the year.

**Response**: This is done. The sentence now reads as follows: “For example, our results indicate higher transmission peak in May/June time, so perhaps the November spray rounds may have artificially bigger impacts due to the natural decline in transmission observed during these months of the year.” See lines 273-275.

On line 274 I would simply add – “but were not available.”

**Response**: This is done. The sentence now reads as follows: “Finer intervals like weekly data would help to improve our estimates but were not available.” See lines 278-279.

Perhaps add as a limitation that you assume no change in the level of insecticide resistance to either product throughout the time-series (if you have susceptibility bioassays for the region perhaps you could corroborate this assumption showing or referencing those).

**Response**: This is done by adding a fourth limitation as follows: “Fourth, we assume no change in the level of insecticide resistance to either product throughout the time-series. We did not have susceptibility bioassays for the study region to corroborate this assumption.” Lines 291-293.

I think there is a policy suggestion from your analysis that you could reasonably add to the discussion –

This work highlights well the variability in the length of time that the active ingredient deployed during a spray campaign may have, even for the same product within a single setting. Real-time surveillance of trends in clinical incidence could be used to help advise strategy teams on when to re-deploy the IRS intervention, ensuring rotation of products as guided by insecticide resistance management policies [you can ref WHO].

**Response**: Thank you so much for this suggested discussion. A paragraph has been added in the discussion as follows: “This work highlights well the variability in the length of time that the active ingredient deployed during a spray campaign may have, even for the same product within a single setting. Real-time surveillance of trends in clinical incidence could be used to help advise strategy teams on when to re-deploy the IRS intervention, ensuring rotation of products as guided by insecticide resistance management policies(14).” Lines 280-284.

The only other comment I have is that spray campaigns sometimes take months to deliver to a community, so perhaps it is worth noting the length of time, in these communities, that it took to complete delivery of each spray campaign and the ultimate percentage of households that were then covered in each spray round. Does this make a difference to the predictions?! Perhaps beyond the scope of the current paper as it is nearly ready to accept, but something to consider in future perhaps.

**Response**: A sentence showing length of time in months it took to complete each round of IRS is added under the study setting section as follows: “The length of time in months it took to complete delivery of each spray campaign were 1.9 for round 1, 1.3 for round 2, 0.8 for round 3, 1.0 for round 4, 2.5 for round 5, and 1.3 for round 6.” See lines 109-111. Unfortunately, we could not estimate the ultimate percentage of households that were covered because data on household listing were not available. Yes, it’s likely that the duration of completing each round of IRS could affect the prediction. This analysis will be considered in the future.
