# Supplementary material for: Estimating the optimal interval between rounds of indoor residual spraying of insecticide using malaria incidence data from cohort studies
Source: PLoS One. 2020 Oct 23;15(10):e0241033. doi: 10.1371/journal.pone.0241033 (PMC7584202; doi:10.1371/journal.pone.0241033)
Supplement: Attachment — Submitted filename: Reviewer Comments PLoS one 2020.docx [file pone.0241033.s004.docx]

Article: PONE-D-20-03773

Title: Estimating the timing interval for malarial indoor residual spraying: a modelling approach

Summary: This is an interesting analysis of clinical incidence data tracking a cohort of under 10-year olds living in a high transmission setting that has implemented IRS. During Phase I, the study explores the use of bendiocarb sprayed approximately bi-annually. During phase II, the study explores annual implementation with Actellic 300®CS. The analysis is robust, and this is an interesting method to determine the window between spray rounds. However, I have some major concerns for the authors to consider prior to acceptance for publication.

Major concerns

1. The authors do not mention the natural seasonality of mosquitoes, how their density may change in the absence of chemical intervention, or whether these changes subsequently drive any seasonality in clinical incidence. Please could the authors comment on how this was accounted for in the statistical model as an increase in cases (the optimal point) could still indicate a working IRS product if the cases increase at a slower rate than expected in the absence of said product at specific times of the year. Were there any data on mosquito vector densities to corroborate the impact on incidence is driven by the IRS impact?

2. How was clinical incidence monitored in the trial? Were rapid diagnostic tests used to confirm positivity? This is important because if the presence of fevers are used to indicate malaria positivity (the authors note symptoms, but do not specify what these are for this study) then ‘true’ seasonality may be dampened because of other infections causing fever that are mis-diagnosed to be malaria. Please comment to clarify.

3. As all participants also received an ITN on enrolment, how were the impacts of these interventions decoupled from the impact of the IRS products? How were the effects adjusted for later rounds of IRS when the ITN efficacy would have waned and adherence to ITN use may have diminished?

In addition, were ITNs redistributed at any point through the study? If not, is the impact from Actellic even greater because it acts with older ITNs – potentially used less by the community and almost certainly working less effectively because the active ingredient has waned. It is critical to comment or account for this effect.

4. Given that malaria cases are so sensitive to the local ecology for any given transmission season, could the authors comment on the uncertainty in their suggested interval for spray rounds and whether they can infer anything from the statistical model about how this window may vary season to season, year to year or across any other boundary. Is it possible to make any sort of prediction for other settings using this framework? The authors note that uncertainty will be considered in future work but this should be included here. Could you infer uncertainty from the 90% credible intervals of the Bayesian simulations carried through from the splines? Or use the mean +/- 1.96*SE to estimate some uncertainty for the spline predictions shown in Fig 3?

Is there any sense in fitting the estimate across rounds for each chemistry? Rather than after each round separately to try and infer a more generalised estimate for this interval between spraying? Could the authors source data from other sites to refit the model with additional data which would enable the uncertainty to be better captured and results to be generalised which would be more useful for guiding policy decisions.

Minor suggestions

The title might be more informative, perhaps try:

“Estimating the interval between indoor spraying of residual chemistries from clinical incidence data”

“Optimising the interval between residual spraying of insecticides indoors in a high transmission setting”

The term ‘modelling’ is fairly broad so I think the title could better reflect the work if this were altered.

Line 98: ‘diving’ should be ‘dividing’

Line 221: ‘long-acting’ should be ‘longer-acting’

Line 230 and 231: should read ‘concurs’ and ‘concur with’ not ‘conquers’ or ‘conquer with’
