# Supplementary material for: Estimating the optimal interval between rounds of indoor residual spraying of insecticide using malaria incidence data from cohort studies
Source: PLoS One. 2020 Oct 23;15(10):e0241033. doi: 10.1371/journal.pone.0241033 (PMC7584202; doi:10.1371/journal.pone.0241033)
Supplement: Attachment — Submitted filename: Reviewer Comments PLoS one 2020_R2.docx [file pone.0241033.s008.docx]

Review

Article: PONE-D-20-03773R2

Title: Estimating the Optimal Interval between Rounds of Indoor Residual Spraying of Insecticide Using Malaria Incidence Data from Cohort Studies

Summary: The work tracks clinical incidence data for a cohort of 6-month to 10-year olds living in a high transmission setting that has implemented IRS. During Phase I, the study explores the use of bendiocarb sprayed approximately bi-annually. During phase II, the study explores annual implementation with Actellic 300®CS. The draft is improved on the previous versions and the previous comments are addressed well. I have a few minor comments to add and a couple of suggestions to pass on to the authors.

**Introduction**

There is a reference for the statement in lines 68-70: (<https://malariajournal.biomedcentral.com/articles/10.1186/s12936-020-3102-6>)

This sentence, starting line 70, is not quite correct as thatch is mentioned twice – please correct:

“A study in Zimbabwe showed that bendiocarb remains active for up to 8 weeks with 96% mortality for mosquitoes on thatch, and 20 weeks with 74% mortality for mosquitoes on mud compared to 100% for mosquitoes on thatch (10).”

Similarly the following sentence needs to be adjusted slightly for clarity:

“While in Madagascar, bendiocarb was shown to maintain 80% mosquito mortality for up to 5 months post spraying (11).”

Throughout this second paragraph of the introduction, efficacy is used without defining exactly what is meant. Perhaps state that a mortality inducing effect where more than 80% of susceptible or local mosquitoes are killed in a bioassay test on the sprayed surfaces is considered effective by WHO guidelines [ref]. (*In my opinion, if sprays are killing mosquitoes at a less potent level they may well still have robust impact on incidence – but this is not the WHO definition at the moment!)* Otherwise you could stick to quantifying this effect by stating that the mortality inducing effect on local mosquitoes remained above 80% for xx weeks as noted by the referenced studies.

One interesting part of your study is that your tested outcome is a clinical one. Testing IRS on mosquito mortality alone forces us to make an assumption about how reduced mosquitoes may lead to reduced clinical incidence. Your approach allows you to test the durability of the spray campaigns on the clinical outcome. I think this is worth highlighting, either as a sentence in the introduction or discussion.

It is also worth noting that malaria transmission in Uganda is mostly perennial, so even though there are slight seasonal peaks, transmission continues all year making the durability of the IRS product an important consideration. This helps understand the analysis too because the 6-month periods can be more consistently compared than would be the case in an area that had more seasonal transmission.

**Discussion**

Line 259 should be ‘concurs’ not ‘concur’

On line 264 perhaps say ‘may leave communities unprotected’ rather than may render IRS ineffective

Lines 269- Perhaps say ‘due to the natural decline in transmission observed during these months of the year’.

On line 274 I would simply add – “but were not available.”

Perhaps add as a limitation that you assume no change in the level of insecticide resistance to either product throughout the time-series (if you have susceptibility bioassays for the region perhaps you could corroborate this assumption showing or referencing those).

I think there is a policy suggestion from your analysis that you could reasonably add to the discussion –

This work highlights well the variability in the length of time that the active ingredient deployed during a spray campaign may have, even for the same product within a single setting. Real-time surveillance of trends in clinical incidence could be used to help advise strategy teams on when to re-deploy the IRS intervention, ensuring rotation of products as guided by insecticide resistance management policies [you can ref WHO].

The only other comment I have is that spray campaigns sometimes take months to deliver to a community, so perhaps it is worth noting the length of time, in these communities, that it took to complete delivery of each spray campaign and the ultimate percentage of households that were then covered in each spray round. Does this make a difference to the predictions?! Perhaps beyond the scope of the current paper as it is nearly ready to accept, but something to consider in future perhaps.
